# Fatigue in children using motor imagery and P300 brain-computer interfaces

**DOI:** 10.1186/s12984-024-01349-2

**Published:** 2024-04-24

**Authors:** Joanna RG. Keough, Brian Irvine, Dion Kelly, James Wrightson, Daniel Comaduran Marquez, Eli Kinney-Lang, Adam Kirton

**Affiliations:** 1 Departments of Pediatrics and Clinical Neurosciences, Cumming School of Medicine, University of Calgary, Calgary, AB Canada; 2 Faculty of Medicine, University of British Columbia, Vancouver, BC Canada

**Keywords:** Brain-computer interface, Pediatrics, Fatigue, Electroencephalography, Cerebral palsy, P300, Motor imagery

## Abstract

**Background:**

Brain-computer interface (BCI) technology offers children with quadriplegic cerebral palsy unique opportunities for communication, environmental exploration, learning, and game play. Research in adults demonstrates a negative impact of fatigue on BCI enjoyment, while effects on BCI performance are variable. To date, there have been no pediatric studies of BCI fatigue. The purpose of this study was to assess the effects of two different BCI paradigms, motor imagery and visual P300, on the development of self-reported fatigue and an electroencephalography (EEG) biomarker of fatigue in typically developing children.

**Methods:**

Thirty-seven typically-developing school-aged children were recruited to a prospective, crossover study. Participants attended three sessions: (A) motor imagery-BCI, (B) visual P300-BCI, and (C) video viewing (control). The motor imagery task involved an imagined left- or right-hand squeeze. The P300 task involved attending to one square on a 3 × 3 grid during a random single flash sequence. Each paradigm had respective calibration periods and a similar visual counting game. Primary outcomes were self-reported fatigue and the power of the EEG alpha band both collected during resting-state periods pre- and post-task. Self-reported fatigue was measured using a 10-point visual analog scale. EEG alpha band power was calculated as the integrated power spectral density from 8 to 12 Hz of the EEG spectrum.

**Results:**

Thirty-two children completed the protocol (age range 7–16, 63% female). Self-reported fatigue and EEG alpha band power increased across all sessions (*F*_(1,155)_ = 33.9, *p* < 0.001; *F* = 5.0_(1,149)_, *p* = 0.027 respectively). No differences in fatigue development were observed between session types. There was no correlation between self-reported fatigue and EEG alpha band power change. BCI performance varied between participants and paradigms as expected but was not associated with self-reported fatigue or EEG alpha band power.

**Conclusion:**

Short periods (30-mintues) of BCI use can increase self-reported fatigue and EEG alpha band power to a similar degree in children performing motor imagery and P300 BCI paradigms. Performance was not associated with our measures of fatigue; the impact of fatigue on useability and enjoyment is unclear. Our results reflect the variability of fatigue and the BCI experience more broadly in children and warrant further investigation.

**Supplementary Information:**

The online version contains supplementary material available at 10.1186/s12984-024-01349-2.

## Background

Cerebral Palsy (CP) is a group of heterogeneous movement disorders and the leading form of lifelong disability. In Canada alone it is projected that there will be nearly 100,000 individuals living with CP by 2031 [1]. In its most severe form, quadriplegic CP, individuals lack muscular control in all limbs, the head, neck and trunk [2, 3]. Children with quadriplegic CP are often cognitively capable but have extremely limited ability to move or speak, a condition analogous to “locked-in syndrome” [4]. Intellectual disability is thought to occur in approximately 50% of children with CP, with higher rates for those with spastic quadriplegic CP [5]. However, many individuals with this severe movement disorder are highly aware and capable. It is important to consider that cognitive impairments are likely overestimated, and deficits may be compounded by decreased opportunities for meaningful environmental interaction [6]. Children with CP are an exemplar group to consider given the nature of the syndrome and the prevalence within the population however, children with other neurodevelopmental disabilities such as spinal muscular atrophy in addition to other neurological conditions acquired in childhood may experience similar barriers. There is a critical need to develop alternative means of connection, communication, and play for children who experience this type of cognitive motor dissociation, and brain-computer interface (BCI) technology has the potential to meet this need [7, 8].

A BCI translates a user’s brain activity to directly control an effector device such as a game, wheelchair, or computer [9]. BCI research is dominated by studies in adult populations [10], but recently it has been demonstrated that children, both typically developing and those with CP, can learn to control BCI systems [11–14]. Factors by which the young brains of children can achieve optimal BCI performance are unstudied but must be identified to improve such potentially life-changing interventions. In adults, operating a BCI has been associated with increased self-reported fatigue [15, 16], and fatigue may negatively impact BCI signal feature detection and operator performance [17]. Fatigue is also an important functional consideration for children with CP [18, 19]. Increased fatigue levels following BCI use have been anecdotally observed in our clinical BCI program working with children on a regular basis but the effects of fatigue on BCI performance remain undefined in children.

The feeling of fatigue is common and universal, yet hard to define. For this research, fatigue will be defined as “a sensation of tiredness, often accompanied by alterations in behaviour or performance, which can arise from sustained performance in a cognitively or physically demanding task” [19–21]. In addition to self-reported metrics and performance changes, adult BCI studies have identified electroencephalography (EEG) biomarkers of fatigue [22, 23] most notably increases in alpha band power [24, 25].

BCIs can increase the ability of children with quadriplegic CP and locked-in-syndrome to actively participate in daily life. Research is needed to optimize such neurotechnology specifically for children. An exploration of fatigue is not only useful for user-centered design of pediatric BCI systems but may contribute to the generation of early fatigue detection tools, mitigation strategies, and adaptive BCIs in the future. The aims of this study were to assess (a) if children experience greater fatigue using a BCI compared to an active non-BCI task as well as to assess any differences in development of fatigue between two commonly used BCI paradigms, motor imagery (MI) and visual P300, and (b) if resting-state neurophysiological measures of fatigue correlate with self-reported fatigue in typically developing children. We hypothesized that (a) fatigue would be greater in the BCI tasks compared to the control and that the MI-BCI would be more fatiguing than the P300-BCI, and (b) that there would be an increase in EEG alpha band power as self-reported fatigue increased.

## Methods

### Participants

Thirty-seven typically developing children, recruited from the community via HICCUP, the Health Infants and Children Clinical Research Program [26], participated in this study (median age 10, mean 9.8, range 6–16, 58% female sex, 54% identify as women). Inclusion criteria were age 6–17 years, absence of any neurological or neurodevelopmental conditions or medications, and informed consent/assent. The study protocol was approved by the Conjoint Health Research Ethics Board, at the University of Calgary (ID: REB22-0044).

A priori power analysis was performed using the power.mmrm function in the longpower R package [27] with an estimated one-tailed standardized effect size of 0.7, alpha = 0.05, beta = 0.8. An N of 33 participants was required to detect this effect.

### Protocol

**Fig. 1:**
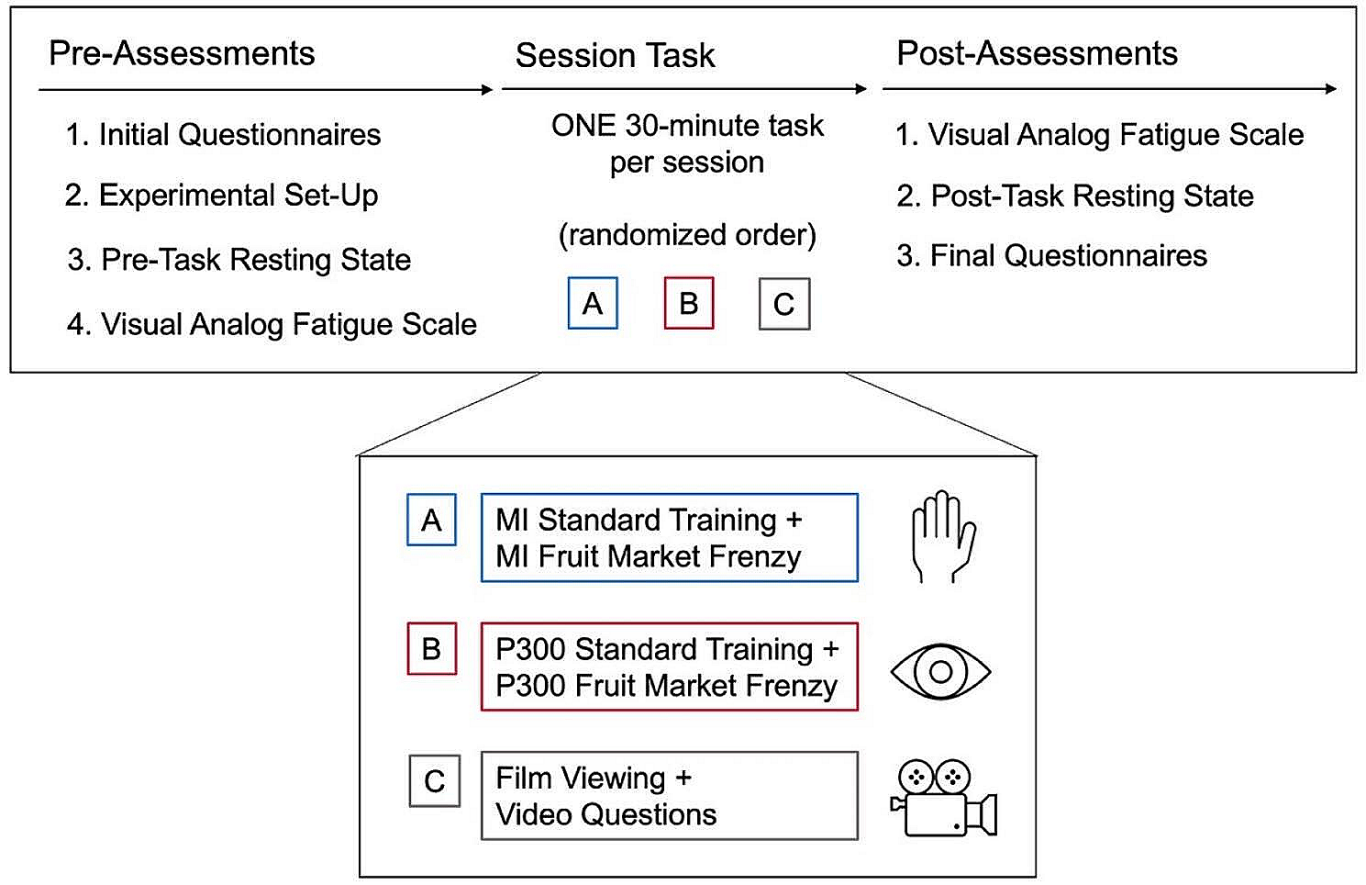
Protocol Schematic for All Three Sessions. Session tasks were balanced using a Latin square design. Sessions lasted 60 to 90 min. MI = motor imagery

Participants attended three sessions on separate days at the Alberta Children’s Hospital BCI4kids laboratory: two BCI sessions (one MI task and one P300 task), and an additional film viewing session (control condition). The session order was balanced using a Latin square design. The length of time between sessions was at least 24 h. The longest time between session was 53 days with an average of 14 days between each session. The protocol was identical in all three sessions before and after the unique session task. A protocol schematic is outlined in Fig. 1.

### Experimental set-up and BCI system

Participants were seated in a chair in front of a 27” LG27GL850 (LG Corporation, Seoul, South Korea) computer monitor for the duration of each session. The monitor has a refresh rate of up to 144 Hz, and a pixel response time of 1 ms. The DSI24-C (Wearable Sensing, San Diego, USA) system was used for EEG monitoring. The DSI24-C is a child size dry electrode EEG headset with 19 active EEG electrodes. Data from this headset was sampled at 300 Hz. The DSI24-C has electrodes with preconfigured positioning. Electrode static positions: Fp1, Fp2, F7, F3, Fz, F4, F8, T3, C3, Cz, C4, T4, T5, P3, P4, T6, O1, O2. Fpz was the ground electrode, and the left ear clip (A1) was used as reference to substantially reduce the occurrence of electrical and motion artifacts [28]. The DSI streamer software was used to establish connection during set up. For optimal connection, we aimed to have impedance between 0.1-5MΩ, RMS Noise < 20µV, and baseline DC shift < +/- 5000µV. Lab streaming layer (LSL) [29] was used with a custom Unity (San Francisco, USA) application and python backend during the data collection.

### Questionnaires

Before each session, participants responded to predefined questions regarding potential factors affecting performance including, mood, sleep quality and duration, exercise, and time since their last meal in the Preliminary BCI Assessment. They also completed the PedsQL™ Multidimensional Fatigue Scale (MFS; acute scale, version 3.0) [30]. During the first session, participants also completed the Edinburgh Handedness Inventory. Assessments and questionnaires at the end of each session were an adapted Pediatric Motivation Scale [31], the Child-Adapted NASA-Task Load Index (TLX) [32], and the BCI Tolerability Assessment. A visual analog scale for fatigue (VASF) was included in the Preliminary BCI assessment and in the BCI Tolerability Assessment. Additional fatigue assessments were done with the VASF immediately before the BCI or film task and immediately after (see timing in Fig. 1) as well as in 5-minute intervals throughout the session task. In the film viewing sessions, to ensure participants were paying attention to the film, they were asked additional questions about the content of the film. They were informed at the start of the task that there would be questions.

### Resting state (eyes-open)

RS was completed immediately before and after the session task (pre-task and post-task). This was a 2-minute EEG recording period where participants were instructed to relax their body and face, refrain from speaking and moving, clear their mind, and look at a target on the computer screen.

### BCI applications

#### MI Application Training

**Fig. 2:**
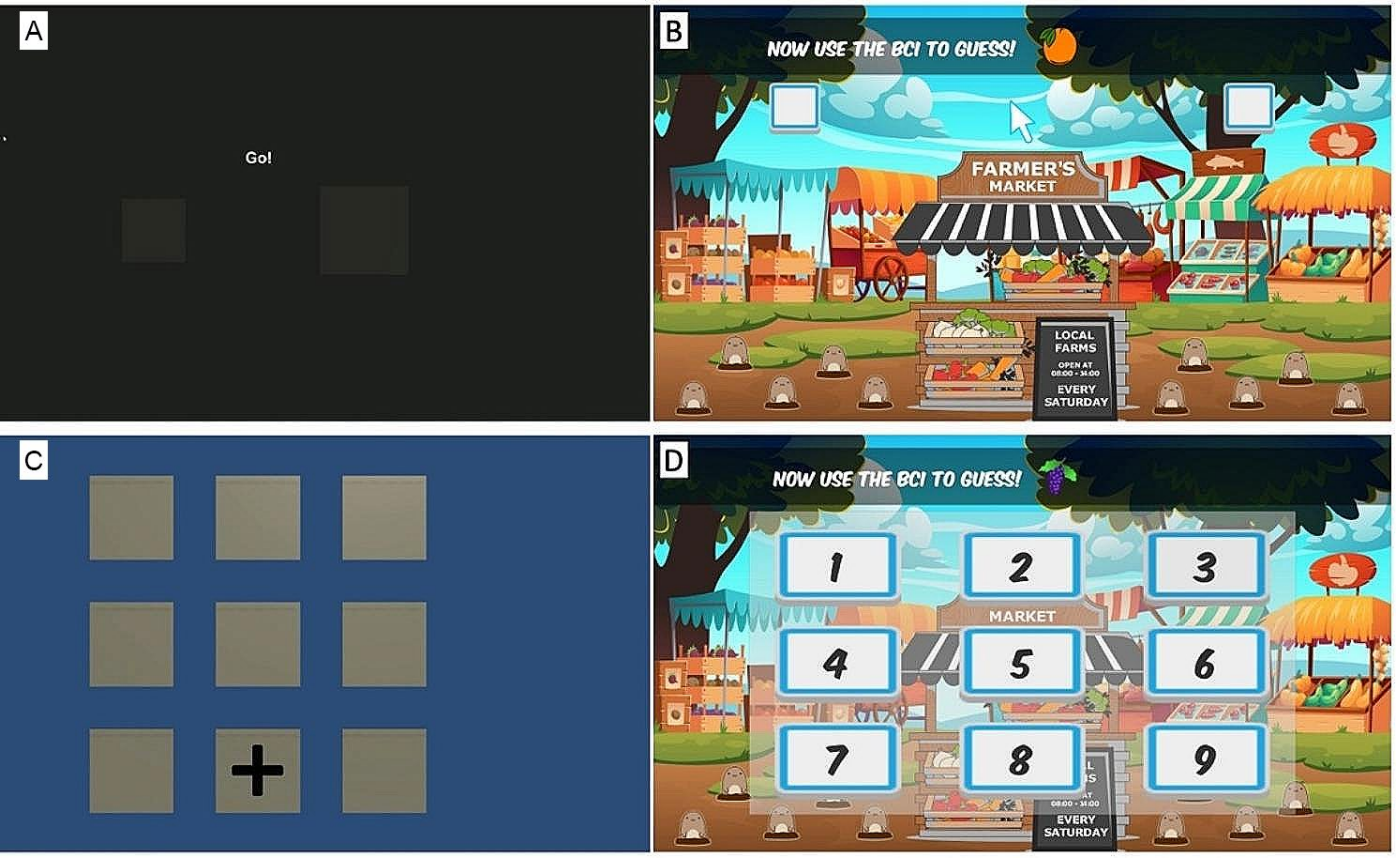
BCI training and fruit marking frenzy game applications. **A**. Motor imagery training scene. **B**. Motor imagery game scene. **C**. P300 training scene. **D**. P300 game scene

Participants trained the classifier for 5 min. The MI task was an imagined hand squeeze. During the standard training, participants were presented with two boxes side by side on a black screen (see Fig. 2A). Boxes alternated between being the standard size or a larger size. The larger box was the “train target”. They were asked to perform their imagined squeeze with the hand on the same side as the “train target” (i.e., if the right box was larger, they performed right hand motor imagery). After one trial on each hand, they were given feedback in the form of the boxes changing colour. The right box became brighter orange when right hand MI was detected while the left box became brighter blue when left MI was detected. Figure 2A – top left panel - depicts the MI training task. EEG was collected for 12 s with 2 s windows and there was a 6 s break between trials. MI alternated between left and right hand for the entire training period. There was a total of 18 training selections. This training design was modified from mindBEAGLE SMR training [33] based on in lab pilot testing.

For MI classification, each 2 s window during MI was filtered with a 5th order Butterworth bandpass filter with corner frequencies of 5 and 30 Hz. A covariance matrix was calculated for each 2 s window. Tangent space mapping was used to get a feature vector from the covariance matrix [34]. A logistic regression classifier was trained using the tangent space feature vectors with the corresponding window labels. This classifier, retrained from scratch every two training selections, was used to provide feedback to the user. k-fold cross validation was used in MI training to help avoid overfitting.

#### P300 application training

Participants trained the classifier for 5 min. During the standard training, a 3 × 3 grid of grey boxes was displayed on the computer screen, as seen in Fig. 2C – bottom left panel. The participants were instructed to focus their attention on the box where a target cursor would appear. They were instructed to only focus on the target box as they all began to flash red in random order. Boxes would turn red for 100 ms followed by a 75 ms pause before the next flash. Participants were instructed to count how many times the box they were focused on flashed. After a random flashing sequence of 15 single flashes per box, there would be a 2 s pause, before the target would appear in a new box, and they were instructed to shift their attention and repeat the above instructions. There was a total of 9 training selections (all the grid boxes). This training design was modified from Guger and Colleagues [35].

Throughout the training period, for each flash a window in the EEG, including the 600 ms from immediately when the flash goes on, was saved. All windows were then filtered with a 5th order Butterworth bandpass filter with corner frequencies of 0.1 and 15 Hz and then were ensemble averaged to yield one window per box per trial. The window corresponding to the trials target box was labeled target and the rest are labeled non-target. XDawn covariance matrices were calculated from each ensemble average windows. Tangent space mapping was used to get a feature vector from the covariance matrix. Shrinkage Linear Discriminant Analysis (sLDA) was used to classify between target and non-target. The combination of XDawn, Riemannian geometry, and sLDA was based on recommendations in [36]. This trained a binary classifier. To make a selection, the posterior probability that each box is the target was calculated. The box with the greatest posterior probability was selected. k-fold cross validation was also used for P300 training.

#### Common MI/P300 game - fruit market frenzy

This application was designed in-house using Unity (San Francisco, USA). Participants watch a fruit stand animation with moles bobbing in and out of holes in the ground. The moles were throwing around common fruit on the screen. During the MI version, children were asked to determine if there was more of a certain kind of fruit (ex. apples) being thrown from the left of the right of the screen. They would verbalize their answer and then try and enter it using the BCI, doing their imagined hand squeeze action to move a cursor in the center of the screen to the left or right (Fig. 2B). A net of four classifier selections in one direction were required to make a selection. Thus any selection in the opposite direction needed to be compensated for with an equal number of correct selections. This continued until a selection was made. During the P300 version, the same animation was used and children were asked to count the number of a certain kind of fruit being thrown around. After verbalizing, they then entered their answers on a 3 × 3 grid of numbers 1 through 9 by attending to the number they were trying to select as the board went through a random flash sequence, 15 flashes/square, similar to the P300 training (Fig. 2D). Windows corresponding to each possible selection were ensembled averaged and classified. The window with the highest posterior probability of being the target was selected.

Participants played these games for 25 min or until they requested to stop. Participants were free to terminate at any time for any reason. While the Fruit Market Frenzy Game involved counting, the “correct” answer for the BCI and the feedback that was given to participants was based on what they shared verbally to the individual collecting the data. This person entered the participants verbal answer with the mouse before participants went on to make their BCI selection.

### Control condition

*Viewing a film (*https://www.youtube.com/watch?v=Mik9iDj0seY*)*: Participants watched 30 min of a YouTube film about nature and animals titled *The Most Amazing Master Builders in the Animal Kingdon* (linked above). Participants were free to terminate the video at any time for any reason.

### Data analysis

Primary outcomes included self-reported VASF and the RS alpha band power pre- and post-task. Electrodes with impedance greater than 5MΩ were removed. Across 106 sessions a total of 13 electrodes were excluded on a session-by-session. If an electrode was excluded it was just for the one session due to noise. The integrated power spectral density (iPSD) was calculated using Welch’s method with 10 s windows and 5 s overlap between windows [37]. iPSD was calculated for the entire 120 s RS window for frequency resolution < 0.01 Hz. The trapezoidal rule for integration was used to get absolute power of the classic alpha band (8–12 Hz). Alpha band power values greater than 99 µV^2^/Hz were excluded from analysis.

Composite scores for motivation, workload, and BCI tolerability questionaries were calculated for use in statistical models described below. Motivation was divided into two groups based on the range of participant scores. Scores ranged from 26 to 45. There was a higher (36–45) and a lower (26–35) motivation group that participants were categorized into. Workload was dived into four groups based on the range of the scale from 0 to 100. The performance question was excluded. Low to high workload groups were split as follows: 0–24, 25–49, 50–74, 75–100. Tolerability measure used in the statistical models was the pain question rated from 0 to 5. Participants were grouped into low to no discomfort (0–2) and higher discomfort (3–5). BCI metrics, accuracy, precision, recall, and the confusion matrix were recorded from the BCI training periods. Overall game performance was also calculated as number of trials correct/number of trials total in the Fruit Market Frenzy Game. Due to limited sample size, raw age values were divided into an upper and a lower half by date of birth.

### Statistical analysis

Statistical analyses were performed using R programming language (version 4.1.2) using the Jamovi software application (2.3.21.0), and the rm_corr library in python (3.8.13). Linear mixed modeling fit with restricted maximum likelihoods was used to assess differences in the two primary outcomes. The main models for both self-reported fatigue (i.e. VASF) scores and alpha band power included session and time (pre/post) as factors and participants as the cluster variable. The random factor was participant intercepts. Age, sex, session length, time of the session, MFS score, workload score, headset tolerability, and motivation score covariates/factors were included in the secondary models and model quality was compared using the Akaike information criterion (AIC) and examination of the fixed effects omnibus tests. Two additional models for VASF included (a) age and sex as factors, and (b) age and sex as factors and MFS score as a covariate. One additional model for alpha band power included motivation score and time of day as covariates.

In both primary outcome models, significant fixed effects omnibus tests underwent further post-hoc analysis with holm correction. Normality of the data was confirmed with visual inspection of Q-Q normality plots and density histograms. For the alpha iPSD model, outliers were also identified by visual inspection of residual histograms. To ensure outliers did not unduly influence statistical models, models were rerun with outliers removed. The threshold for rejecting the null hypothesis was *p* < 0.05. Error in measurement or absolute reliability was calculated for the main outcome measures, alpha band power and VASF score. The calculation methods can be found in Hopkins work [38]. Repeated measure correlations were performed to assess relationships between both primary outcomes as well as relationships between primary outcomes and secondary outcomes.

## Results

Three of the 37 children consented for participation in this study were subsequently realized to have a diagnosis of attention deficit hyperactivity disorder, and their data was excluded from analysis. One participant chose to withdraw from the study part way through their first session and another individual did not fit our headset and thus could not participate. This left a final study population of 32 participants. Two of the final 32 had remote prior concussion. Three participants had a MFS score of less than 50 consistent across sessions.

### Self-reported fatigue

**Fig. 3:**
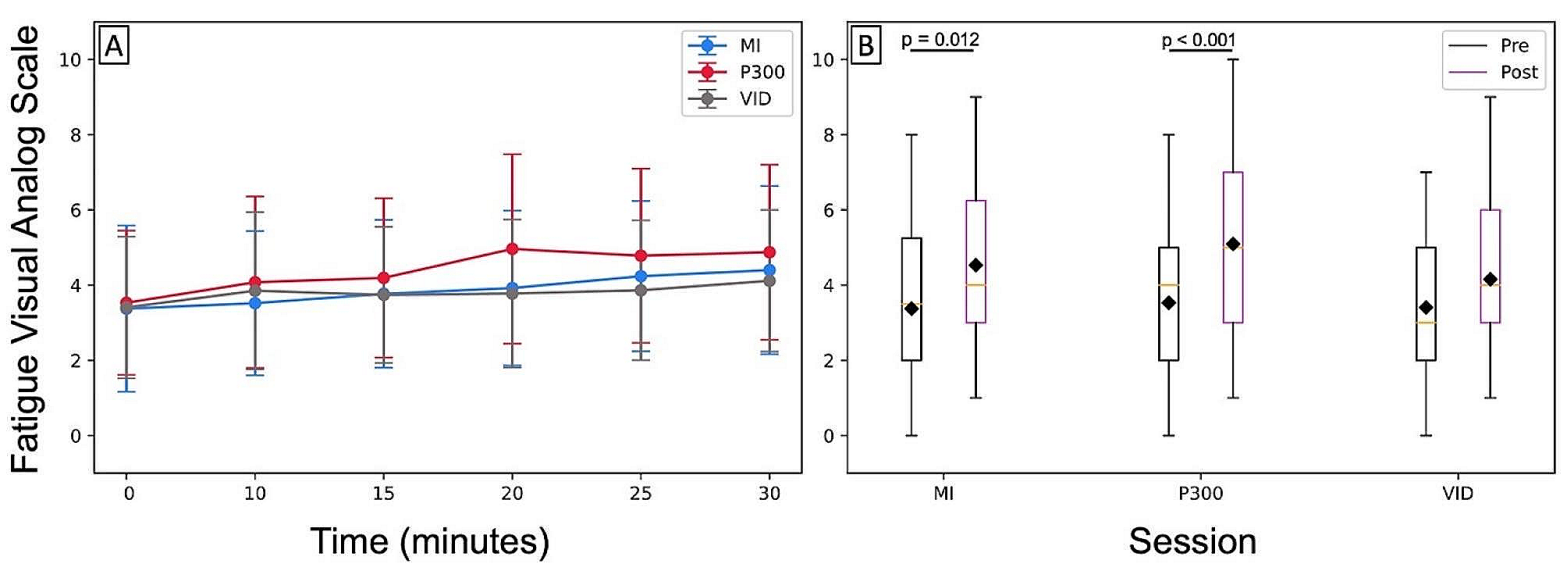
Self-Reported Fatigue. **A**. Fatigue visual analog scale values reported across the session at time 0,10,15,20,25, and 30 min. **B**. Fatigue visual analog scale values pre-task and post-task for each session. Post-task was at time 30 for most participants, but for those who did not complete the full protocol post-task is not at time 30. Boxplot lines indicate quartiles. Orange lines indicate the median and black diamonds are sample mean. Bars are plus or minus 1.5x the interquartile range. MI: motor imagery; VID: video

There was a main effect of time on self-reported fatigue (*F*_(1,155)_ = 33.9, *p* < 0.001), but no main effect of session (*F*_(2,155)_ = 2.5, *p* = 0.086) or interaction effect of session and time (*F*_(2,155)_ = 1.4, *p* = 0.250) on self-reported fatigue. The estimated marginal mean VASF score across all sessions was 3.4 (95% CI 2.8–4.1) pre-task and 4.6 (95% CI 4.0-5.2) post task. The mean VASF pre- and post-task for each session is in Table 1 (Fig. 3). The statistical model AIC value was 748.

An additional model with inclusion of age and sex (model 2) as factors demonstrated an interaction effect of age and time (*F*_(1,140)_ = 4.5, *p* = 0.036). The younger half of participants had a larger change in VASF from pre to post across all sessions. The addition of MFS score into the model with age and sex (model 3) found an effect of MFS scores (*F*_(1,129.2)_ = 6.6, *p* = 0.012). Those with MFS scores greater than one standard deviation from the mean had lower VASF values and those with scores one standard deviation lower than the mean had higher VASF values. Further information on secondary models is included in supplemental material Table 1; Fig. 1 in Additional File 1. There was no effect of time of day, session length, workload, motivation, headset tolerability, or BCI game performance on VASF.

### Electroencephalography alpha band power

**Fig. 4:**
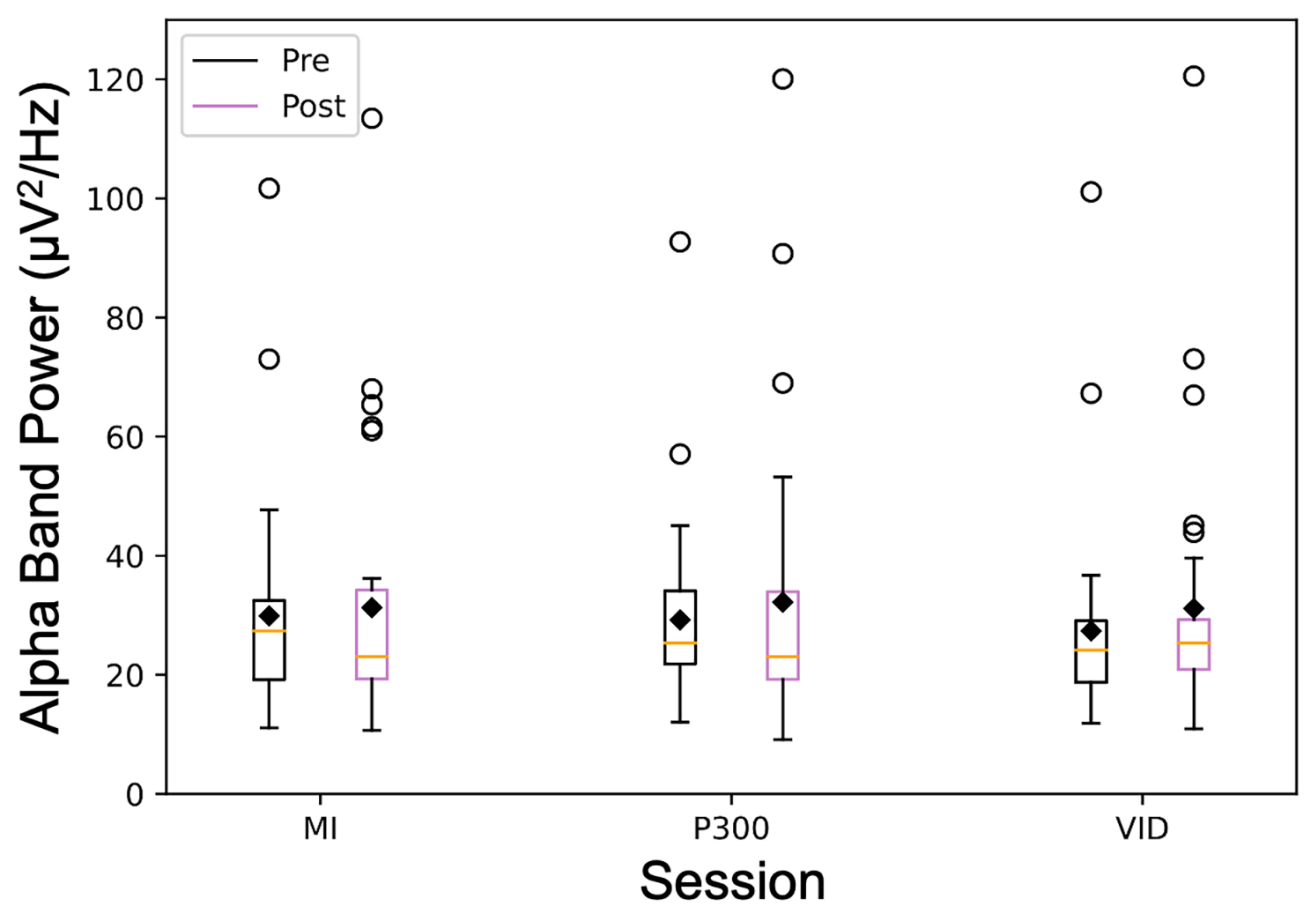
Alpha Band Power. pre-task resting-state and post-task resting state alpha band power for each session. Boxplot lines indicate quartiles. Orange lines indicate the median and black diamonds are sample mean. Bars are plus or minus 1.5x the interquartile range. MI: motor imagery; VID: video

Before statistical analysis, EEG alpha band power values were excluded from 6/96 sessions. There was a main effect of time on alpha band power (*F*_(1,149)_ = 5.0, *p* = 0.027) but no effect of session (*F*_(2,149)_ = 1.2, *p* = 0.319) or interaction effect (*F*_(2,149)_ = 0.2, *p* = 0.842). The estimated marginal mean across all session was 29.4 µV^2^/Hz (95% CI = 22.6–36.1) during pre-task RS and 32.2 µV^2^/Hz (95% CI = 25.5–39.0) during post-task RS. The mean alpha band power pre- and post-task for each session is in Table 1 (Fig. 4). The AIC value was 1447. There was no effect of age, sex, session length, time of day, motivation, workload, headset tolerability, or game performance on the alpha band power change.

**Table 1:** Fatigue visual analog scale values and alpha band power pre- and post-task

Session	Time	Self-Reported Fatigue (10 pt scale) (95% Confidence Interval)	Alpha Band Power (µV^2^/Hz) (95% Confidence Interval)
MI	Pre	3.4 (2.6–4.1)	30.0 (22.9–37.1)
	Post	4.5 (3.8–5.3)	31.8 (24.6–39.0)
P300	Pre	3.5 (2.8–4. 3)	30.3 (23.2–37.5)
	Post	5.1 (4.3–5.8)	33.5 (26.4–40.7)
Video	Pre	3.4 (2.7–4.2)	27.8 (20.6–34.9)
	Post	4.2 (3.5–4.9)	33.1 (24.2–38.5)

Legend: Values are estimated marginal means. MI: motor imagery

### BCI performance

**Fig. 5:**
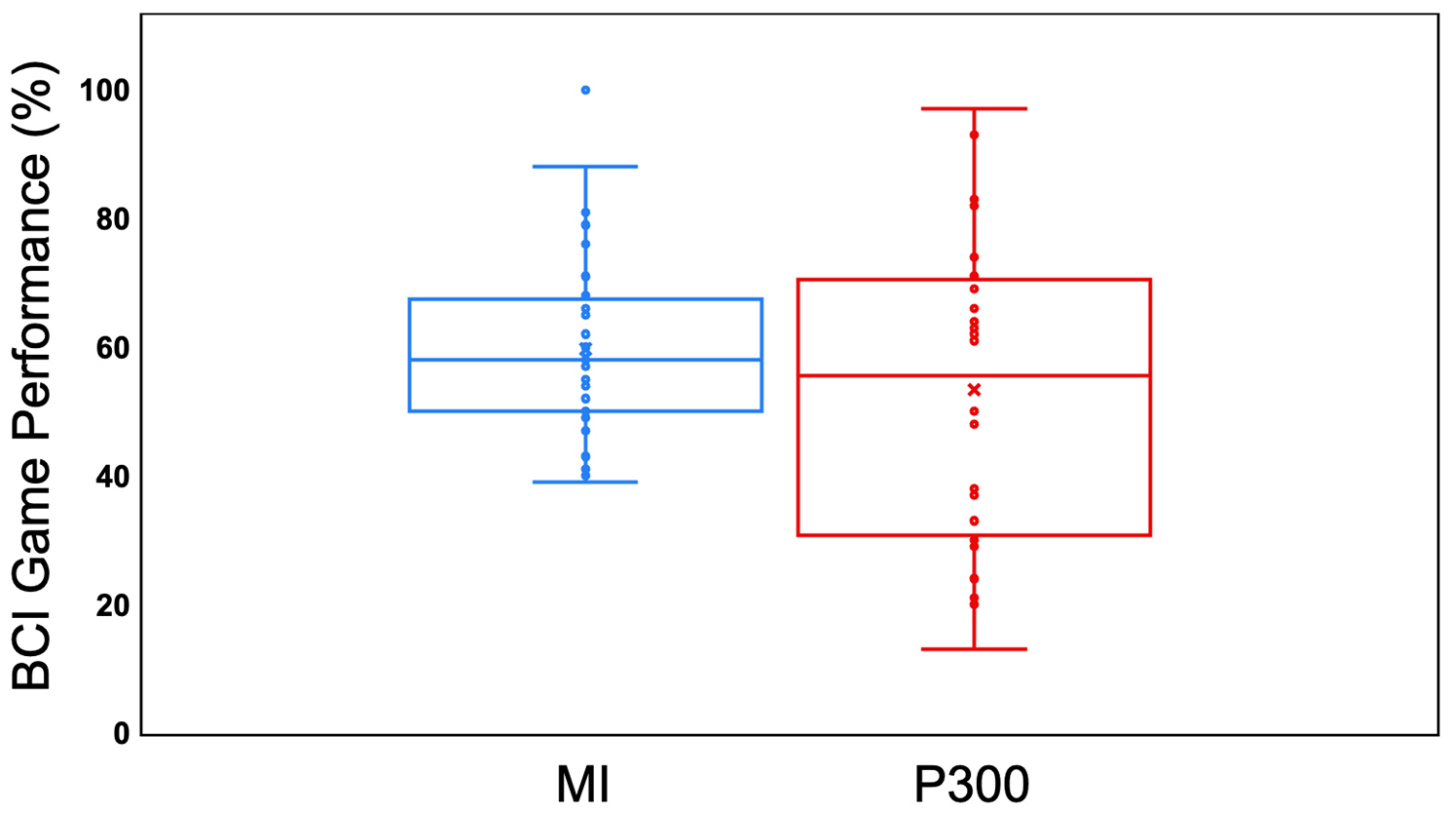
BCI Fruit Market Frenzy game performance. Performance calculated by total trials correct/total trials for each session. Boxplot lines indicate quartiles, and all participant data is plotted. Bars are plus or minus 1.5x the interquartile range. BCI: Brain-computer interface; MI: motor imagery

BCI training scores are shown in Tables 2 and 3 and BCI Fruit Market Frenzy performance is in Table 2; Fig. 5. For reference, during the Fruit Market Frenzy game, MI classification is binary with chance accuracy of 50%. P300 is a one in nine selection with a chance accuracy of 11%. Regardless of training scores, all participants played the Fruit Market Frenzy game. During MI training, only 3 individuals achieved a training accuracy above 70% (14 above 60%), but 7 individuals achieved greater than 70% during the MI Fruit Market Frenzy game following training (12 above 60%). All participants achieved training accuracy above 70% for P300 training. Eight individuals achieved this percentage in the P300 Fruit Market Frenzy game (16 above 60%). Comparisons for P300 training and game are more difficult because the training is a binary classification between 2 classes, target and non-target, and the game online accuracy is comparing posterior probabilities of that classification for each of the 9 boxes (chance = 11%). MI game performance ranged from 39 to 100%. P300 game performance ranged from 13 to 97%. Across the participants there was no significant increase or decrease in performance over time.

**Table 2:** BCI training and game performance scores

Performance Outcome	Mean	Range	95% Confidence Interval	
Training accuracy				
MI	0.61	0.43		0.57–0.64
P300	0.94	0.15		0.93–0.95
Training precision				
MI	0.61	0.44		0.57–0.64
P300	0.91	1.0		0.80–1.01
Training recall				
MI	0.61	0.54		0.57–0.65
P300	0.48	1.00		0.36–0.59
Game performance				
MI	0.60	0.61		0.56–0.64
P300	0.55	0.84		0.48–0.61

Legend: Training accuracy is the ratio of correct predictions over total predictions made by the model. Training precision is the ratio of true positives over all positive classifications (true and false positives). Training recall is the ratio of true positives over all actual positives (true positive and false negatives). Training scores computed by game performance was calculated by total trials correct/total trials. MI: motor imagery

**Table 3:** Confusion matrix for motor imagery and P300 training

	Requested Selection				
**Participant Selection** **P**	Motor Imagery Mean			Motor Imagery Range	
		Target	Non-Target	Target	Non-Target
	Target	21	33	6–35	19–48
	Non-Target	33	21	24–48	3–36
	P300 Mean			P300 Range	
		Target	Non-Target	Target	Non-Target
	Target	5	0	0–9	0–3
	Non-Target	4	72	0–9	27–69

Legend: Confusion matrix contains all classifications made in training. From left to right top to bottom of each quadrant they are true targets, false targets, false non-targets, and true non-targets

### Questionnaires: mood, workload, motivation, and tolerability

**Fig. 6:**
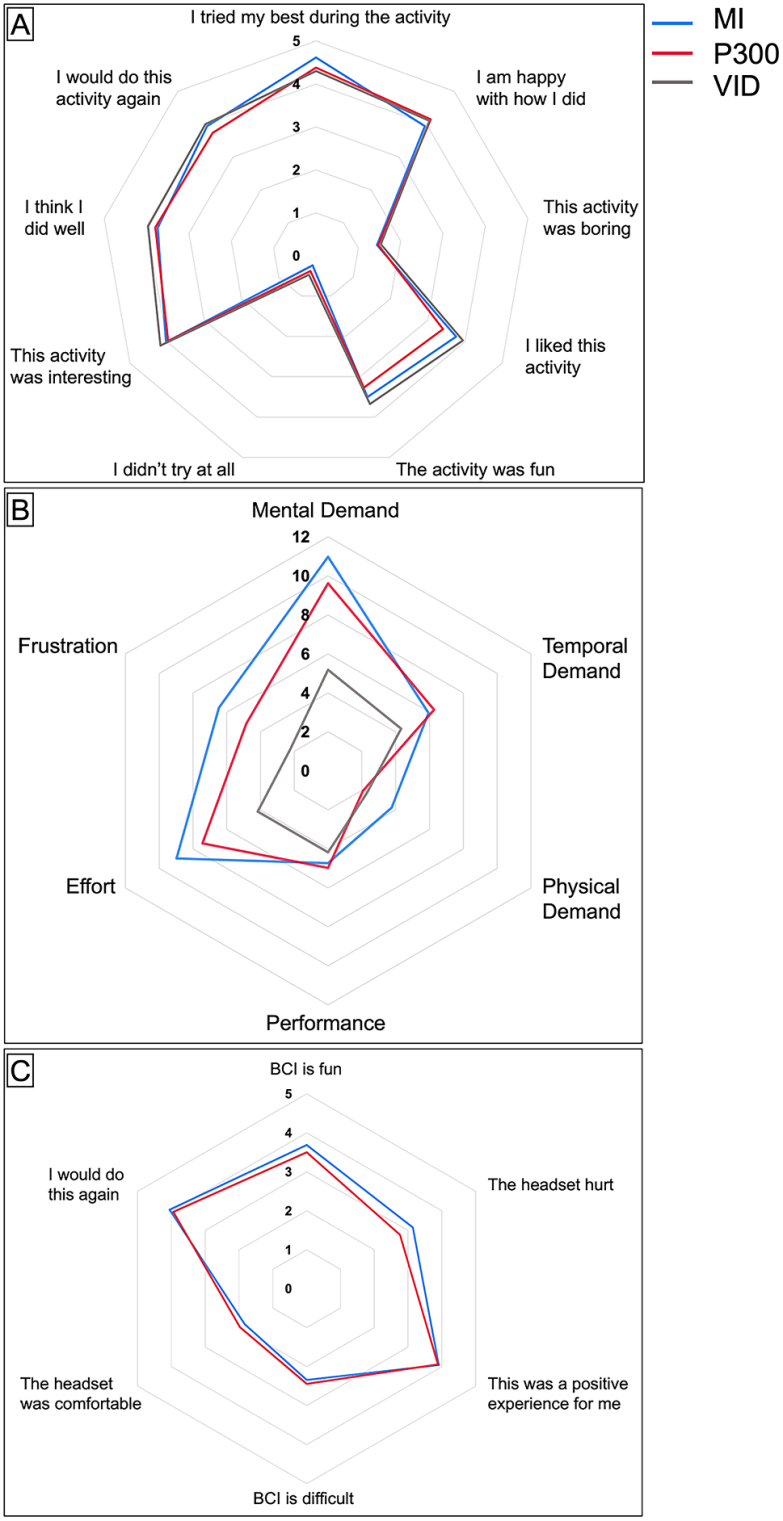
Self-Reported Post-Task Questionnaire Metrics. **A**. Pediatric Motivation Scale from not true at all (0 pts) to definitely true (5 pts) **B**. Child Adapted NASA Task-Load Index from low demand (0 pts) to high demand (20 pts). **C**. BCI Tolerability Scale from not true at all (0 pts) to definitely true (5 pts). BCI: Brain-computer interface; MI: motor imagery; VID: video

During 69% of the 96 sessions, participants came in with positive moods: ‘excited’, ‘chill’, ‘pleasant’, ‘calm’, ‘happy’, ‘content’, ‘comfortable’, ‘lively’, ‘fulfilled’. In the other 31% of sessions, participants came in with more neutral or negative mood states: ‘neutral’, ‘sleepy’, ‘tired’, ‘nervous’, ‘hurt’, and ‘bothered’. Ratings from the Pediatric Motivation Scale were similar across all sessions (Fig. 6A; Table 4). Mental demand, temporal demand, effort, and frustration were all similar between both BCI sessions and tended to be higher compared to the control session (Fig. 6B; Table 4). Physical demand was often higher in the MI BCI session compared to the P300 BCI session and the control (Fig. 6B; Table 4). BCI tolerability ratings were similar between MI and P300 sessions (Fig. 6C; Table 4). The majority of participants found the headset uncomfortable (72% during MI, 59% during P300, 53% during control). There was an increase in the number of individuals who reported discomfort across the three sessions, 56% during the first session, 59% during the second session, and 72% during the third session. 22% of participants did not report discomfort in any session. 25% of participants were unable to complete the whole protocol due to discomfort part way through the sessions.

**Table 4:** Average questionnaire scores

Questionnaire	Mean	Median	Range	95% Confidence Interval
Motivation Scale (possible range = 0–50)				
MI	29.3	29.5	13	28.4–30.3
P300	28.4	29.0	20	27.4–29.4
Video	30.3	31.0	13	29.2–31.3
NASA – Task Load Index (possible range = 0–120)				
MI	41.4	43	71	36.4–46.4
P300	35.8	31	59	31.6–40.0
Video	22.2	20	57	18.6–25.8
BCI Tolerability Scale (possible range = 0–30)				
MI	19.2	20	11	18.4–19.9
P300	18.4	19	14	17.7–19.2

Legend: MI: motor imagery

Repeated measures correlation analysis revealed a negative correlation between change VASF and motivation (*r* = -0.28, *p* = 0.022). The VASF change was also positively correlated with the self-reported mental demand (*r* = 0.28, *p* = 0.027) but not temporal demand or physical demand. Change in VASF was correlated with effort (*r* = 0.25, *p* = 0.044). Mental demand, temporal demand, and physical demand correlated with frustration (*r* = 0.45, *p* < 0.001; *r* = 0.28, *p* = 0.023; *r* = 0.35, *p* = 0.005 respectively). Motivation negatively correlated with frustration (*r* = -0.33, *p* = 0.01). Measured game performance was negatively correlated with self-reported performance evaluation (*r* = -0.47, *p* = 0.006). There was no correlation between change in self-reported fatigue and change in alpha band power (*r* = 0.08, *p* = 0.532).

## Discussion

The aim of this research was to better understand the effect of different BCI paradigms on self-reported fatigue and EEG biomarkers in children. In all conditions, there was an increase in self-reported fatigue post-task compared to pre-task. The difference was small, roughly a 1.5 pt difference on a 10 pt scale. There was also a small increase in alpha band power in all conditions. Contrary to our hypothesis, our sample was unable to demonstrate differences in self-reported fatigue or RS EEG alpha band power related to BCI tasks between watching a video, P300, and MI tasks. There was also no correlation between the change in self-reported fatigue and alpha band power as described in adults. We demonstrated that short periods of both MI and P300 BCI operation increase self-reported fatigue in children, but our results do not support the use of EEG generic alpha band power during pre-and post-task RS as a sensitive biomarker for fatigue.

The increase in VASF during the P300 session was greater than the increase during the video session. There were also significant increases in VASF from pre- to post-task in the MI and P300 sessions but not the video session (Fig. 3). Prior adult BCI studies looking at self-reported fatigue during BCI use have found increases of above 3 pts on a 10 pt scale [15, 39], and between 1 and 3 pt on a 20 pt scale [16]. The standard error of measurement of the VASF scale for our data was 1.1 pts. Calculated with this error, the minimum detectable change is predicted at 3.2 pts on the 10 pt scale, much larger than what we observed. Our hypothesis regarding self-reported fatigue for this study was built off unpublished work in our lab (Kelly et al. in review). This work demonstrated variable fatigue with multiple BCI paradigms and MI-BCI was more fatiguing than P300-BCI with a roughly 1.5 pt VASF increase. In contrast we found that P300-BCI sessions had the greatest increase in VASF, P300-BCI was not associated with any increase in VASF in this unpublished work (Kelly et al. in progress).

This previous pediatric BCI study by Kelly et al. (in review) was a different experimental set up than the present study, and any number of differences may have impacted user experience and fatigue. In Kelly and Colleagues work P300 and MI tasks were completed on the same day in a randomized order and tasks were 8–20 min in length. The training, the task, and the BCI hardware/software were also not consistent between our two studies. In the prior study, the age of participants was slightly higher with a mean age of 11.3. The primary research question was about BCI control in children, with self-reported fatigue as a secondary outcome. Even though the task length was longer in the current study, present results from self-reported fatigue indicate P300 was fatiguing even at 10 and 15 min. We found similar numbers of participants had reported an increase by one or more points on the VASF in the P300-task and the MI-task by these time points (10 min: 8 participants in P300, 6 in MI; 15 min: 13 in P300, 12 in MI). Particularly in the P300 session, however, younger participants had a VASF increase of over two points while older participants did not even have a one-point increase at this time. The slightly older participants in Kelly and Colleagues work may have contributed the difference in fatigue with P300 that we observed. The VASF score change observed across time in all sessions was greater for the younger children in this present study. We found that older children and adolescents were better able to handle 30-mintues of a BCI task without significant fatigue and generally did not find the video session fatiguing (supplemental Fig. 1, Additional file 1). It is also likely that having the tasks in the same study session, as Dion and Colleagues did, had a different impact on self-reported fatigue than having the tasks on separate days. Additional pediatric BCI fatigue studies considering these many variables are required.

Day-to-day chronic fatigue was assessed using the acute PedsQL^TM^-MFS before each session in the present study. As expected, children with lower MFS scores (i.e. higher fatigue) reported higher VASF values pre- and post-task. The impact of more chronic and pathological fatigue will be an important consideration for understanding BCI fatigue in children with CP, who typically have higher chronic fatigue [19]. Further pediatric work is needed to draw conclusions on the impact of more significant fatigue at baseline and its impact on BCI performance and behavioural changes. We suggest that simple, validated measures like the PedsQL^TM^-MFS, for which CP-specific versions are also available, be employed prospectively in BCI studies in such clinical populations to better characterize the role of fatigue in performance and other outcomes.

To our knowledge, this was the first trial to investigate EEG biomarkers of fatigue in children during BCI use. While adult studies show promise in tracking these biomarkers and associating them with self-reported fatigue, more work may be needed in children to refine these measures. During analysis some electrodes were excluded due to signal artifacts or noise. This was either from sub-optimal connection at known specified electrode sites or from participants touching an electrode or moving the headset forehead strap. Identification of individualized alpha bands was unsuccessful with existing python algorithms for use with eyes-open RS. Pipelines were applied but resulted in non-physiological interparticipant and intraparticipant variability. Comparison of alpha band power results from the present work to past literature was also difficult due to variable units, use of undefined units, lack of values given, and use of both relative and absolute metrics. From our data, the standard error of measurement of this band power was 5.0 µV^2^/Hz. Calculated with this error, the minimum detectable change is predicted at 13.8 µV^2^/Hz. A change near this magnitude was not detected. Change in alpha band power magnitude in many prior adult studies is significantly larger and often correlates with self-reported fatigue [15, 16, 23]. Prior EEG studies have found that changes in EEG are not apparent until an individual is highly fatigued [40] and perhaps our interventions were not long or hard enough or lacked appropriate difficulty to reach such a threshold. Age-dependent developmental differences in EEG neurophysiology may also have affected our ability to detect fatigue-related changes.

Despite a broad acceptance of EEG band metrics as fatigue biomarkers, there is still inconsistency in the literature. Particularly in research looking at driver fatigue, several studies have found no change in alpha band power or even a decrease in alpha band power [41]. As an alternative metric, EEG entropy has been calculated as a biomarker for fatigue including during sustained attention cognitive tasks [42, 43] and high stress cognitive tasks [44]. In a driving simulator study, changes in sample entropy were more consistent during fatigued states than band metrics [45]. Peng and Colleagues also found that a multiscale entropy metric better distinguish fatigue states than the classic bands during a steady-state visually evoked potential BCI task [41]. Entropy may therefore be a useful measure in children to help avoid difficult individual frequency band calculations or use of generalized bands which may be less accurate [46–49]. Evaluation and comparison of pre- and post-task RS is complex as post-task RS brain activity can be modulated by other elements of the prior task outside of just fatigue [50]. A combination of band metrics, from the frequency domain, and entropy, from the time or the time-frequency domain [51], may be more useful for a deeper understanding of these complexities [50]. Such dual metric analysis should be investigated in children using BCIs.

Performance varied in both the P300 game and the MI game from 100% or nearly 100% to essentially no control at all considering chance accuracies for each paradigm (< 11% in P300; <50% in MI). Interestingly, those who performed better tended to report feeling that they did worse than those who performed poorly although this was purely correlational. Performance did not decline across the BCI sessions. The larger their change in fatigue, the lower participants rated their session enjoyment. Correlational analysis also revealed that children who felt the tasks were more mentally, physically, and temporally demanding also reported being more frustrated. A link between frustration and workload was also noted in end users with motor impairments during BCI gaming [52]. This study had a small sample of four individuals, but interestingly, this association existed for those who had less severe motor impairment [52]. Due to potential EEG changes with frustration, it has been suggested that EEG signal, BCI classification, and performance may be impacted [53]. However, a previous study looking at the performance-frustration relationship did not report significant influences of frustration on performance [54]. Anecdotal reports from an additional study with Amyotrophic Lateral Sclerosis patients also noted that frustration did not seem to impact motivation [55], but we found a negative correlation between frustration and motivation. Psychological factors such as frustration and motivation are clearly influenced by age and development and need to be considered carefully in pediatric populations.

### Limitations

Our calculated but modest sample size is a potential limitation for this work. The present study was not adequately powered to draw conclusions about differences between sessions since changes in both primary outcomes were minimal. This is particularly true given the variation observed in EEG data for our participant. The broad age range (7–16), with inconsistent samples at each age, also presented additional challenges to age-based analysis that was overcome by splitting the sample into just an upper and a lower half by date of birth. Additional limitations include low headset tolerability that reduced the number of our participants completing the full study protocol. Time on task is accepted as a key predictor of fatigue for physical and cognitive tasks [56–59], and this inconsistency introduced more variability into the study. There is a relationship with fatigue and pain or discomfort and while self-reported pain and comfort ratings for the headset were controlled in the statistical models, discomfort is another potentially confounding variable that may have impacted participants attention throughout the study.

Future studies should continue to evolve our understanding of BCI fatigue in children using larger samples, and/or smaller age ranges to overcome challenges of a highly variable population. Studies should also investigate longer durations of BCI use with a system that will be more broadly tolerated by children. It will be important to consider BCI experience and performance as not all children have good BCI control in one session with no prior training. An initial training session may be beneficial to ensure only those with adequate BCI control go forward and are involved with research questions surrounding fatigue. For those with lower BCI performance, future studies will also be needed to investigate predictors of performance as well as strategies to promote BCI learning. A limitation of any task-based study with attention related outcomes is the impact of natural interest variability among participants. Finally, and most importantly, studies on typically developing children should guide the development of similar studies in children with CP, the intended end users of this technology.

## Conclusion

We found that 30 min of BCI task increased self-reported fatigue and the power of the EEG alpha band. There was no correlation between the change in self-reported fatigue and the change alpha band power. Differences in fatigue development across sessions were not clear with our sample. Large variations in children’s experiences with BCI systems including tolerability, motivation, perceived workload, ability to control the applications, and feelings of fatigue were apparent in this study. Future studies are needed to look at longer time-on-task, additional EEG fatigue biomarkers in children, and importantly, to ask similar questions in clinical populations who may use BCI as an access technology.

### Electronic supplementary material

Below is the link to the electronic supplementary material.

Supplementary Material 1: Secondary models from linear mixed model analysis of primary outcomes. Figure of self-reported fatigue data segregated by participant age.

## Data Availability

The dataset supporting the conclusion of the article is available from the corresponding author on reasonable request.

## Abbreviations

BCI: Brain-computer interface
CP: Cerebral palsy
EEG: Electroencephalography
iPSD: Integrated power spectral density
MFS: Multidimensional fatigue scale
MI: Motor imagery
RS: Resting-state
TLX: Task-load index
VASF: Visual analog scale for fatigue
