# Supplementary material for: Fatigue in children using motor imagery and P300 brain-computer interfaces
Source: J Neuroeng Rehabil. 2024 Apr 24;21:61. doi: 10.1186/s12984-024-01349-2 (PMC11040843; doi:10.1186/s12984-024-01349-2)
Supplement: Supplementary file 1 — Supplementary Material 1: Secondary models from linear mixed model analysis of primary outcomes. Figure of self-reported fatigue data segregated by participant age. [file 12984_2024_1349_MOESM1_ESM.docx]

**Supplemental Material**

***Table 1. Secondary linear mixed models for self-reported fatigue***

| **Model** | **AIC** | **Effect(s)** |
| --- | --- | --- |
| **Fatigue Visual Analog Scale** | | |
| Additional Factors:  Sex and Age | 760 | Session (*F*_(2,140)_ = 3.4, *p* = 0.035)  Session-Age (*F*_(2,140)_ = 3.2, *p* = 0.44)  Time-Age (*F*_(1,140)_  = 4.5, *p* = 0.036) |
| Additional Factors:  Sex, Age, MFS | 755 | MFS (*F*_(1,129.2)_ = 6.6, *p* = 0.012) |

Legend: *The primary model for the fatigue visual analog scale included session and time as factors, participant as the cluster variable, and participant intercepts as the random factor. AIC; Akaike information criterion, MFS; PedsQL^TM^ Multidimensional Fatigue Scale.*


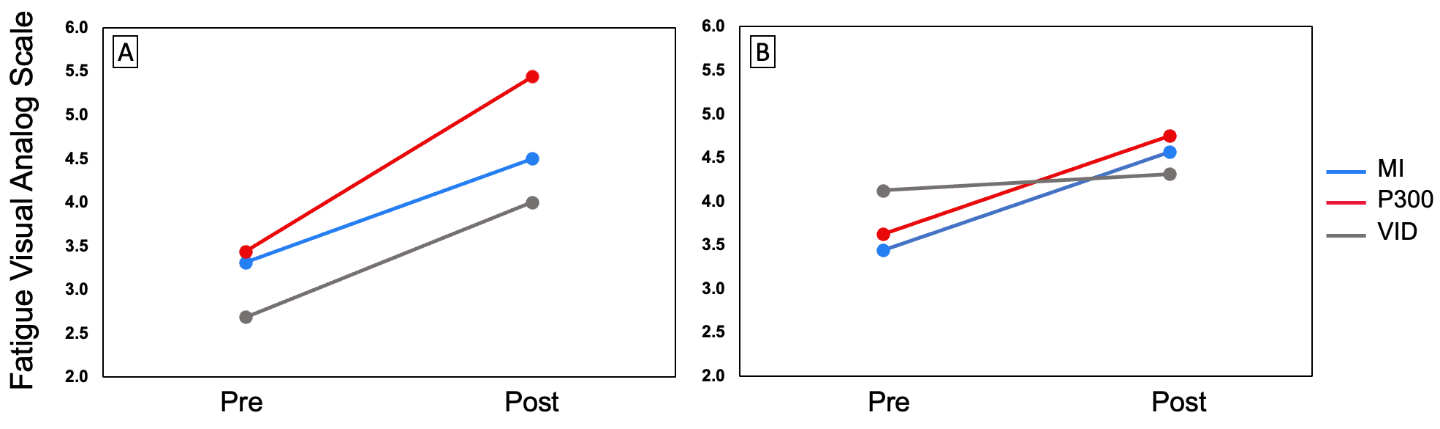
***Figure 1. Visual Analog Scale Values for Self-Reported Fatigue Pre- and Post-Task.*** *A. the younger half of participants, and B. the older half of participants. MI: motor imagery; VID: video.*
